# Comparison of Salvianolic Acid A Adsorption by Phenylboronic-Acid-Functionalized Montmorillonites with Different Intercalators

**DOI:** 10.3390/molecules28135244

**Published:** 2023-07-06

**Authors:** Jun Qian, Jiajia Su, Weihuan Zeng, Yue Wang, Yingyuan Hu, Guoyin Kai

**Affiliations:** 1 Zhejiang Provincial TCM Key Laboratory of Chinese Medicine Resource Innovation and Transformation, Zhejiang Provincial International S&T Cooperation Base for Active Ingredients of Medicinal and Edible Plants and Health, School of Pharmaceutical Sciences, Jinhua Academy, Zhejiang Chinese Medical University, Hangzhou 311402, China; qianjun_1116@163.com (J.Q.); s15951135946@163.com (J.S.); z1437143688@126.com (W.Z.); wy102799@163.com (Y.W.); 2 School of Chemistry and Life Sciences, Suzhou University of Science and Technology, Suzhou 215009, China; huyy@usts.edu.cn

**Keywords:** montmorillonite, polyethyleneimine, silane, salvianolic acid A, adsorption

## Abstract

Due to its success in treating cardio-cerebrovascular illnesses, salvianolic acid A (SAA) from *Salvia miltiorrhiza* is of major importance for effective acquisition. For the adsorption of salvianolic acid, cationic polyelectrolytes, and amino-terminated silane intercalated with phenylboronic-acid-functionalized montmorillonites, known as phenylboronic-acid-functionalized montmorillonites with PEI (PMP) and phenylboronic-acid-functionalized montmorillonites with KH550 (PMK), respectively, were produced. In this paper, detailed comparisons of the SAA adsorption performance and morphology of two adsorbents were performed. PMP showed a higher adsorption efficiency (>88%) over a wide pH range. PMK showed less pH-dependent SAA adsorption with a faster adsorption kinetic fitting in a pseudo-second-order model. For both PMP and PMK, the SAA adsorption processes were endothermic. Additionally, it was clearer how temperature affected PMP adsorption. PMK has a higher adsorption selectivity. This study demonstrates how the type of intercalator can be seen to have an impact on adsorption behavior through various structural variations and offers an alternative suggestion for establishing a dependable method for the synthesis of functional montmorillonite from the intercalator’s perspective.

## 1. Introduction

Cardiovascular and cerebral disorders are reportedly among the leading causes of death in people today [1]. In traditional Chinese medicine, *Salvia miltiorrhiza* has been shown to be effective in treating cardio-cerebrovascular disorders [2]. Tanshinones that are soluble in fat and phenolic acids that are soluble in water are the active ingredients of *Salvia miltiorrhiza* [3]. Everyone is aware that traditional Chinese medicine is frequently prepared with boiling water. Thus, phenolic acid molecules serve as the primary therapeutic agents. Salvinolic acid A (SAA) has been reported to have greater pharmacological action and to be more effective in the treatment and prevention of cardio-cerebrovascular diseases [4]. Remarkably, SAA and its powder injection were given Class I clinical approval by traditional Chinese medicine in 2016 to treat cardiovascular disorders in China.

However, the main source of SAA, *Salvia miltiorrhiza*, only contains about 0.01–0.03% [5], which makes it much more difficult to extract and separate SAA using conventional methods. Additionally, it is challenging to synthesize SAA artificially today. The yield is often low and there are numerous reaction stages, which increases manufacturing costs and makes industrialization challenging [6]. The issues described above may limit the further application and promotion of SAA. Therefore, it is crucial to investigate trustworthy resources for the effective collection of SAA from *Salvia miltiorrhiza*.

For the acquisition of phenolic acids over the last few decades, various processes and methods have been used, including extraction, solid phase adsorption, and precipitation [7,8,9]. Solid phase adsorption has drawn the most attention among them as a result of its adaptability, simplicity, and efficiency [10]. For the separation of SAA, some effective functional materials have been created thus far. For instance, magnetic microspheres modified with amino-terminated cucurbit uril were created to collect SAA [11]. Additionally, it was suggested to use magnetic Fe_3_O_4_ nanoparticles coated in molecularly imprinted polymer for selective SAA capture through a variety of interactions [12].

Due to its inherent chemical stability, significant specific surface area, and affordable price, montmorillonite (MMT), a type of clay mineral with three-lamellar structures made up of a Si-O tetrahedron and an Al-O octahedron, plays a significant role in the field of adsorption [13,14,15]. However, in practice, natural MMT exhibits some drawbacks, including poor selectivity, a slow mass transfer rate, and a small amount of adsorption capacity [16,17,18], which are attributed to the absence of functional groups, narrow interlayer spaces, and a dearth of efficient adsorption sites. It has been shown that intercalation can effectively increase the distance between the layers and introduce some functional groups to boost adsorption or reaction sites for subsequent modification, which might potentially address some of the abovementioned drawbacks [19,20]. Cationic compounds, such as cations [21], cationic polyelectrolytes [16], cationic surfactants [22,23], cationic silanes [24], etc., can be used to intercalate MMT to expand the interlayer space. Among them, cationic polyelectrolytes and cationic silanes commonly have functional groups that can be further modified. Additionally, we discovered that boronic acid and cis-1,2-diol or catechol will react chemically to form cyclic boronic acid ester structures [25,26,27]. Given that the SAA molecule contains many catechol structures, boronic acid can be chosen as a possibility for a functional group to modify MMT and enhance its SAA adsorption capabilities.

**Scheme 1:**
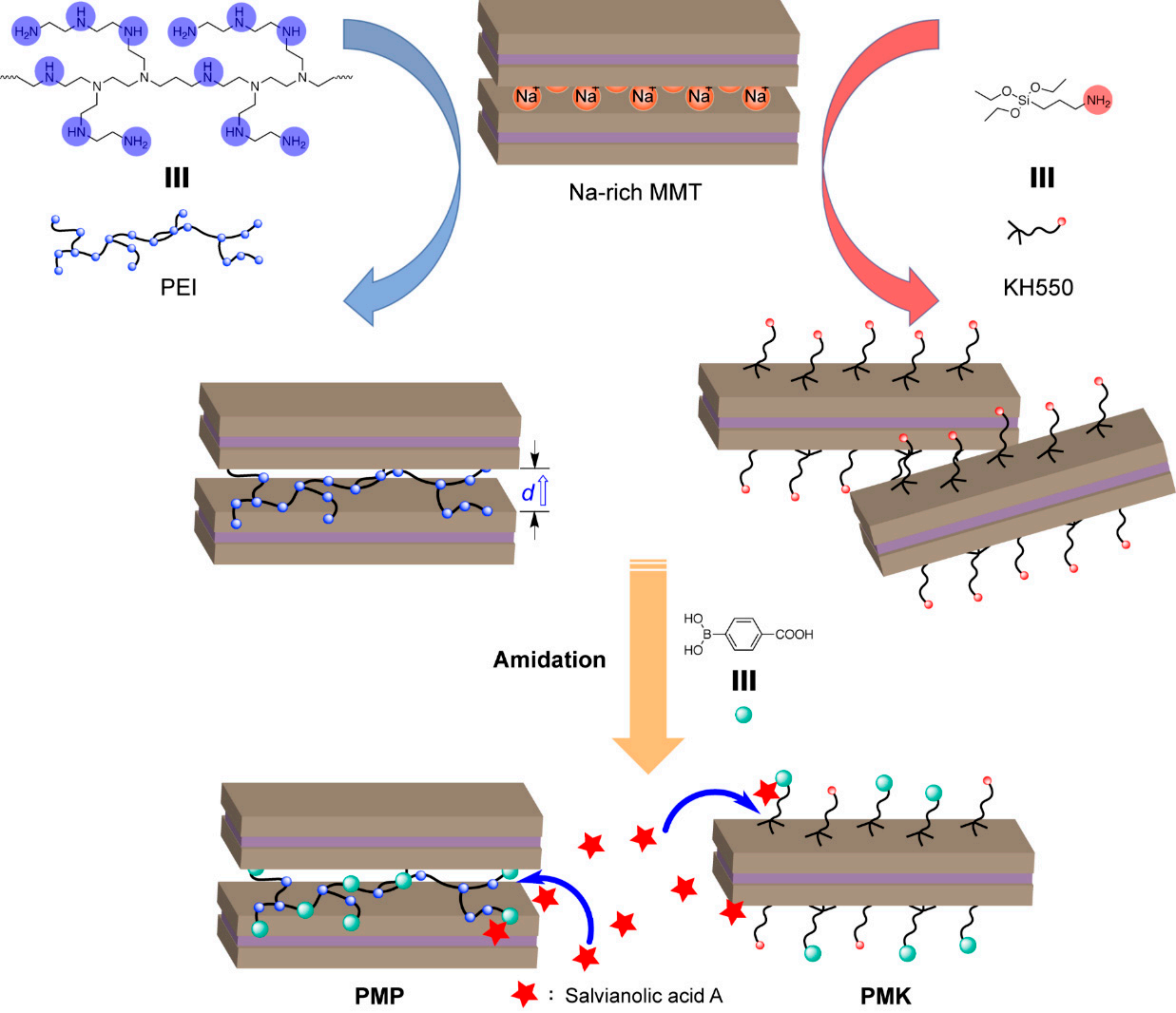
Schematic depiction of the preparation of phenylboronic-acid-functionalized montmorillonites with PEI (PMP) and phenylboronic-acid-functionalized montmorillonites with KH550 (PMK) for the adsorption of SAA.

Herein, branched polyethyleneimine (PEI) and 3-aminopropyltriethoxysilane (KH550) were utilized as intercalators to expand the interlayer distance of MMT, respectively. Then, 4-carboxyphenylboronic acid was reacted with amino groups of MMT-PEI and MMT-KH550 to obtain phenylboronic-acid-functionalized montmorillonites with PEI (PMP) and phenylboronic-acid-functionalized montmorillonites with KH550 (PMK). Scheme 1 presents the synthesis processes. Because it has a lot of readily protonatable amino groups, the cationic polyelectrolyte PEI can intercalate into the MMT interlayers [28]. Similar to PEI, MMT can likewise be intercalated by the terminal amino group of the KH550. Additionally, KH550, as a kind of silane, can engage in a hydrolysis process with the hydroxyl groups of MMT [29]. Structure and adsorption behavior may alter depending on how intercalation occurs differently. The structure, morphology, and subsequent SAA adsorption performance of adsorbents were thoroughly examined to compare the SAA adsorption by PMP and PMK. This study provides a groundbreaking illustration of an in-depth evaluation of SAA adsorption performance using various intercalators. It is expected that a reliable method can be established to prepare a boronic-acid-functionalized montmorillonite composite for the effective adsorption of SAA.

## 2. Results and Discussion

### 2.1. Characterizations of PMP and PMK

#### 2.1.1. SEM Analysis

**Figure 1:**
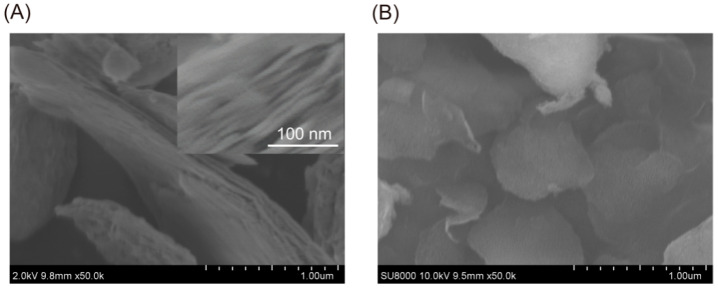
SEM images of (**A**) PMP and (**B**) PMK, scale bar: 1.00 μm. The inset shows the SEM image of PMP at a higher magnification, scale bar: 100 nm.

SEM was used to observe the PMP and PMK morphologies. Figure 1A demonstrates that PMP maintains a layer-by-layer structure like that of natural MMT (Figure S1). The only change in the morphology of PMP is the thickening parts between the layers, resulting from the existence of a polymer. In the case of PMK, intercalation and modification cause the initial sandwich-like structure to disintegrate and transform into several single sheets (Figure 1B). In comparison to PEI, KH550 can produce a more thorough intercalation process. Distinct intercalators that have been used to functionalize phenylboronic acid in MMT result in completely different morphologies, which may have an impact on adsorption behaviors.

#### 2.1.2. XRD Analysis

**Figure 2:**
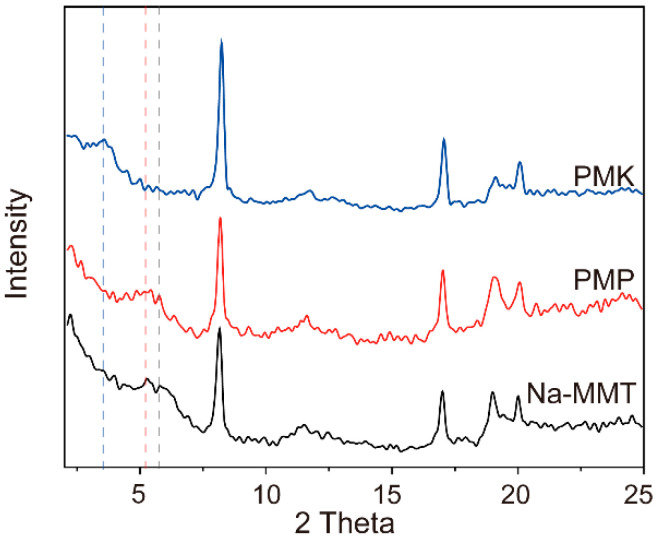
XRD patterns of Na-rich MMT, PMP, and PMK.

XRD was utilized to further characterize the morphological features. In comparison to PMP, the (001) XRD signal of PMK displayed a more pronounced shift after intercalation and modification, indicating a greater change in the interlayer space (Figure 2). In contrast to pristine MMT, the interlayer space of PMK rose after calculation from 14.96 to 25.58 Å, whereas PMP increased to 16.87 Å. The outcomes are consistent with the SEM observation. More thorough intercalation makes the interlayer space of PMK larger. In addition to intercalation, the hydroxyl groups of MMT can be hydrolyzed via a silane such as KH550, which could result in a wider interlayer gap. The combination of intercalation and silane condensation makes the interlayer space of PMK larger than that of PMP.

#### 2.1.3. BET Analysis

BET analysis was carried out to compare the specific surface areas of two MMT composites. The pore diameters and BET surface areas of PMP and PMK differ as a result of the varied degree of intercalation (Figure S2 and Table 1). Intercalation increased the pore size of PMP and decreased its surface area. The original 3D structure of PMK was converted to 2D. Because of this, the surface area of PMK shrank less than that of PMP. Even though PMK has a larger surface area, the effectiveness of adsorption depends more on other factors including the density of adsorption sites and hydrophilicity.

#### 2.1.4. EDX and Zeta Potential Analysis

**Figure 3:**
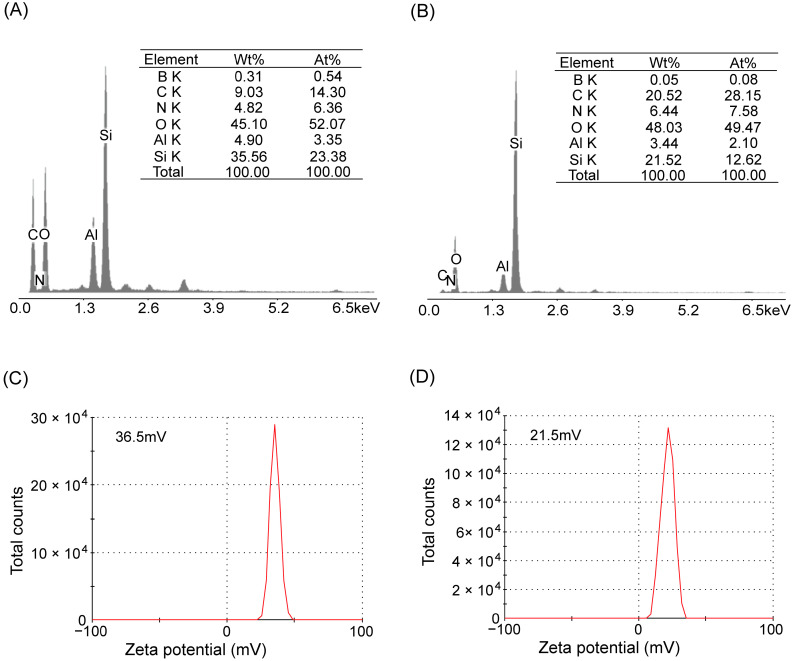
EDX spectra of (**A**) PMP and (**B**) PMK. Zeta potentials of (**C**) PMP and (**D**) PMK in water of pH 7.0 (particle size: <75 μm, sample mass: 0.5%, background electrolyte: deionized water).

Elemental distribution was determined by EDX to better understand the quantities of functional groups on the surfaces of the two MMT composites. According to Figure 3A,B, PMP possesses more functional groups than PMK does, as evidenced by the larger amount of element B in PMP. Additionally, PMP and PMK have zeta potentials of 36.5 and 21.5 mV at pH 7, respectively (Figure 3C,D). The results could be attributed to various numbers of amino groups on the surface of MMT composites. There are still measurable amino groups in PEI after the amidation reaction, and these groups can be protonated to serve as a source of positive charges. The density of amino groups in KH550 is lower than that of PEI. As a result, PMP has a higher zeta potential, which would make it easier to adsorb negatively charged SAA.

### 2.2. Effect of Adsorbent Concentration on SAA Adsorption

**Figure 4:**
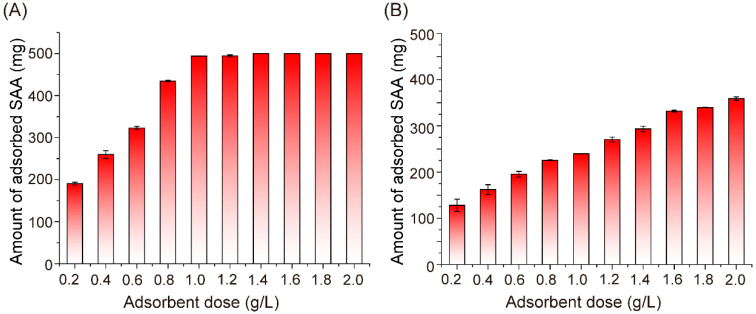
Effect of adsorbent dose on the amount of adsorbed SAA for (**A**) PMP and (**B**) PMK.

To understand the effect of adsorbent concentration on SAA adsorption, the uptake of SAA was investigated as a function of adsorbent concentration ranging from 0.2 to 2.0 g/L at 25 °C for 24 h (Figure 4). As more adsorbent was introduced, more SAA was adsorbed by both PMP and PMK. At a dosage of 1.0 g/L for the adsorbent, the amount of adsorbed SAA for PMP might reach 497.3 mg. In terms of PMK, the adsorption quantity fell short of the equilibrium level within the testing window. The more adsorption sites, including more amino groups, phenylboronic acid groups, and positive charges, might be the cause of the higher adsorption of SAA on PMP. The concentration of the adsorbent was unified as 1.0 g/L to make the comparison of the two adsorbents more understandable.

### 2.3. Effect of pH on SAA Adsorption by PMP and PMK

**Figure 5:**
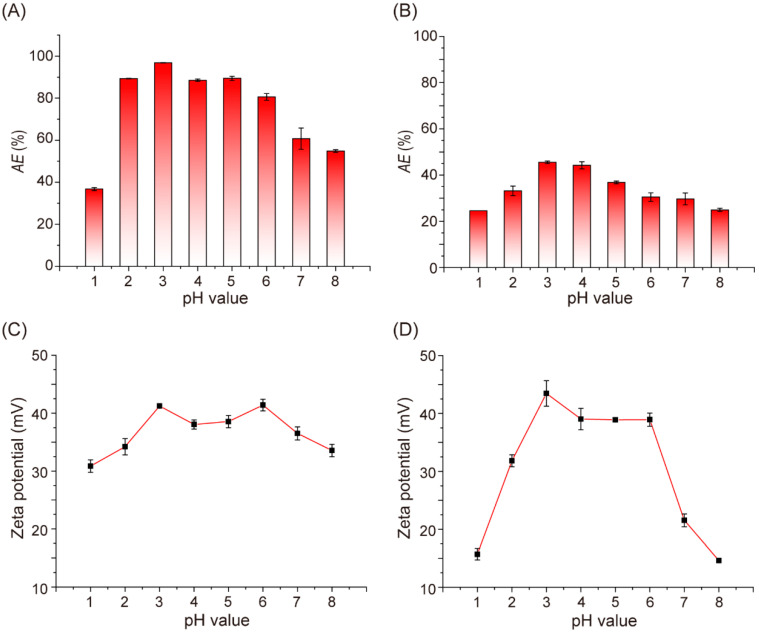
Effect of pH on SAA adsorption by (**A**) PMP and (**B**) PMK. Zeta potentials of (**C**) PMP and (**D**) PMK in various pH conditions.

To compare the differences in adsorption performance between the two composites, the pH of the SAA solution was changed from pH 1 to 8. (Figure 5A,B). With an adsorbent concentration of 1.0 g/L, the SAA adsorption efficiency of PMP could reach over 88% over a wide pH range (2–5) of the solution. Additionally, pH 3 produced the best adsorption results. Regarding PMK, the influence of pH on SAA adsorption by PMK appears to be less clear (24.5–45.5%). The functional group density of PMP and PMK as well as their zeta potentials may be connected to the various adsorption tendencies. The zeta potentials of PMP and PMK in various pH settings were measured to determine the inner link (Figure 5C,D). The change in PMP’s zeta potential was discovered to be apparent. Additionally, the trend change was typically compatible with the variation in SAA uptake under different pH conditions. The outcomes suggested that coulombic interaction might be crucial to the adsorption process. In a pH 3 environment, PMP has a higher zeta potential. Additionally, SAA occurs in an ionic state at pH 3 due to the predicted pKa value being approximately 2.67–2.88. Thus, the stronger electrostatic interaction facilitated the adsorption process. In the case of PMK, the change in zeta potential was more pronounced, but the corresponding change in adsorption performance was less, suggesting that the influence of the functional group was more significant. In other words, the electrostatic force had a negligible effect on the adsorption of PMK. Therefore, the pH of the SAA solution shows less of an effect on the adsorption performance of PMK.

### 2.4. Effects of Contact Time and Temperature on SAA Adsorption by PMP and PMK

**Figure 6:**
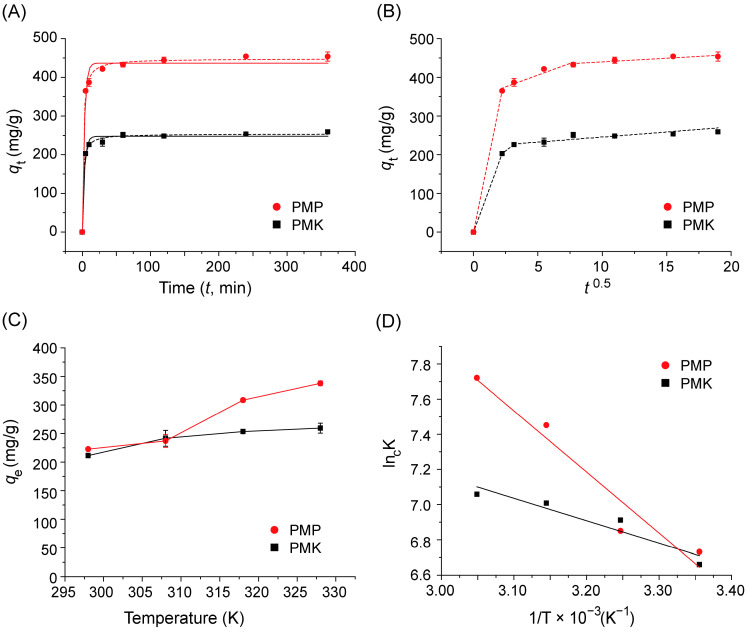
(**A**) Effect of contact time on the adsorption of SAA by PMP and PMK (*c*_0_ = 0.5 mg/mL, pH = 3, *V* = 3 mL, *m*_adosrbent_ = 3 mg, T = 298.15 K). The scattered points stand for experiment data. The solid line and dashed line represent pseudo-first-order and pseudo-second-order model fitting curves, respectively. (**B**) Weber–Morris intragranular diffusion model of SAA adsorption by PMP and PMK. (**C**) Effect of temperature on the adsorption of SAA by PMP and PMK (*c*_0_ = 0.5 mg/mL, pH = 3, *V* = 3 mL, *m*_adosrbent_ = 3 mg), and (**D**) plots of ln*K*_c_ versus 1/*T* for the adsorption of SAA by PMP and PMK.

To gauge the impact of contact time on the two adsorbents, the SAA adsorption kinetics of PMP and PMK were compared. Figure 6A shows that PMP and PMK had a quick adsorption rate and could approach equilibrium in 30 min. The uptake of SAA on PMP was larger than PMK, ascribing to the different amounts of adsorption sites. Pseudo-first-order and pseudo-second-order models, respectively, were used to match the adsorption kinetics of PMP and PMK [30,31,32]. Given the stronger correlation values (*R*^2^) (Table S1), which suggested that chemical adsorption was the rate-limiting step for both adsorbents, a pseudo-second-order model is more appropriate to describe the kinetic profiles of PMP and PMK [33,34]. Interestingly, the *k*_2_ of PMK is higher than that of PMP, demonstrating that PMK has faster adsorption kinetics. The following variables may have an impact on the outcomes: (1) a larger surface area of PMK gave it a higher adsorption rate; (2) the rate-limiting step in the formation of the cyclic boronic acid ester is chemisorption [35]. PMK reached equilibrium more quickly when there were fewer phenylboronic acid groups present.

The Weber–Morris intragranular diffusion model was also examined in order to understand the diffusion characteristics of SAA on PMP and PMK [36]. Figure 6B presents the findings. The three main components of the SAA diffusion processes in PMP and PMK are external adsorption, intra-particle diffusion, and equilibrium. It first took the PMP external adsorption procedure around 5 min. The intra-particle diffusion process then started, lasting for roughly 25 to 30 min. In around 30–35 min, the equilibrium stage can be attained. Compared with PMP, the intra-particle diffusion process for SAA was shorter, which may be ascribed to the 2D structure of PMK. As a result, PMK shows a faster adsorption kinetic in comparison to PMP.

To investigate the effect of temperature on SAA adsorption by PMP and PMK, the adsorption experiments were operated at pH 3 with a 1.0 g/L adsorbent concentration under different temperature conditions (30, 40, 50, and 60 °C). As the temperature rose, more SAA was adsorbed on the PMP and PMK (Figure 6C), indicating an endothermic adsorption mechanism. Surprisingly, compared to PMK, the adsorption of SAA on PMP varied more noticeably with the adsorption temperature. The various contributions of the adsorption process may be responsible for the outcomes. Our earlier research suggests that electrostatic interaction, hydrogen bonds, π-π-stacking interactions between molecules, and covalent bonds all play a role in the adsorption of PMP [17]. As a result, it can be assumed that the adsorption mechanism of SAA on PMK is similar because the functional groups are identical. As is known to all, physical interactions (the first three) are exothermic, while chemical reactions (the latter) are endothermic [35,37,38]. More phenylboronic acid groups are present in PMP, which indicates a larger percentage of chemical reactions. Meanwhile, there was essentially no difference between the PMP and PMK zeta potentials at pH 3. (Figure 5C,D). Therefore, even though PMP’s and PMK’s SAA adsorption processes displayed overall endothermic behavior, the endothermic process for PMP is more pronounced than that of PMK.

Figure 6D depicts the relationships between ln*K*_c_ and 1*/T* for the SAA adsorption by PMP and PMK, and Table S2 lists the associated thermodynamic parameters. Δ*H*^o^ and Δ*S*^o^ for the adsorption of SAA on PMP can be determined to be 28.91 kJ/mol and 152.24 J/mol/K, respectively, which suggests an endothermic and entropy-increasing process. The binding procedure for PMK is the same as for PMP. Δ*H*^o^ and Δ*S*^o^ are calculated as 10.62 kJ/mol and 91.42 J/mol/K, respectively. The SAA adsorptions on PMP and PMK occurred spontaneously in the temperature range of 30 to 60 °C where Δ*G*^o^ can be calculated as negative ones for both PMP and PMK.

### 2.5. Adsorption Isotherms of PMP and PMK

**Figure 7:**
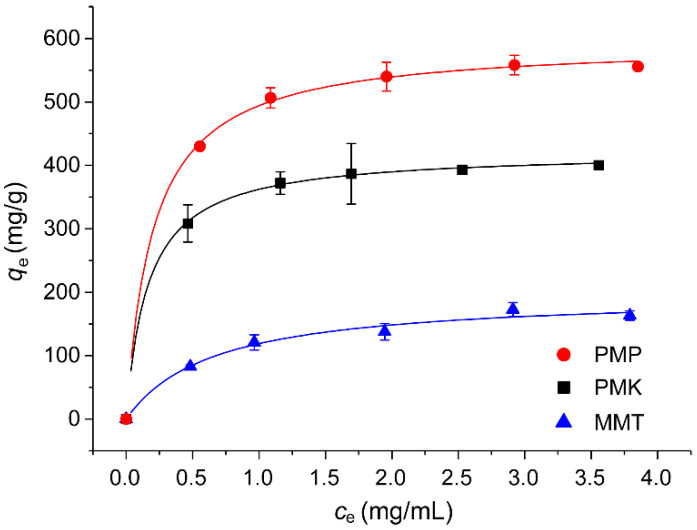
Langmuir isotherm for SAA adsorption on MMT, PMP, and PMK (pH = 3, *V* = 3 mL, *m*_adosrbent_ = 3.0 mg, T = 298.15 K). The scattered points stand for experiment data. The solid lines represent Langmuir isotherm model fitting curves.

To understand the adsorption capacities of MMT, PMP, and PMK for SAA, adsorption isotherm experiments were conducted with varying initial SAA concentrations at pH 3 and 298.15 K. The adsorption profiles were fitted with the Langmuir isotherm model (Figure 7). The adsorption behaviors of the three adsorbents can be described by the Langmuir model well, referring to the comparatively larger correlation coefficients (*R*^2^ > 0.9) (Table S3). Due to the different amounts of functional groups, it can be found that the maximum adsorption capacity (*q*_max_) of MMT, PMP, and PMK for SAA could reach 197.2, 593.2, and 421.2 mg/g, respectively (Table S3). The results may be related to the density of phenylboronic acid groups and positive charges. According to the results in Figure 5, the zeta potentials of PMP and PMK were almost the same at pH 3, indicating that the density of phenylboronic acid groups had a more important role in the adsorption process. To verify this hypothesis, the SAA uptakes of MMT-PEI, MMT-KH550, PMP, and PMK were compared (Figure S3). After the modification of the phenylboronic acid groups, the SAA uptakes showed an obvious increase. The results suggested that the amount of phenylboronic acid groups had an impact on the adsorption capacity of the functional materials.

### 2.6. Selectivity of PMP and PMK

**Figure 8:**
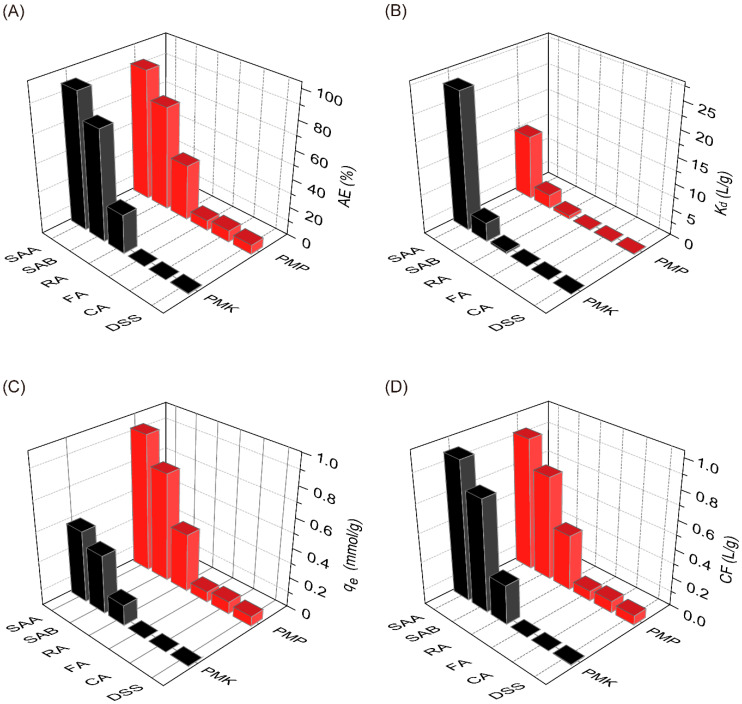
Effects of competing compounds on (**A**) *AE* (%), (**B**) *K*_d_ (L/g), (**C**) *q*_e_ (mmol/g), and (**D**) *CF* (L/g) of PMP and PMK (PMP: *c*_0_ for each salvianolic acid = 1.0 mmol/L, pH = 3, *V* = 3 mL, *m*_adosrbent_ = 3 mg, T = 298.15 K; PMK: *c*_0_ for each salvianolic acid = 0.5 mmol/L, pH = 3, *V* = 3 mL, *m*_adosrbent_ = 3 mg, T = 298.15 K).

To assess the selectivity of the two adsorbents, the effects of competing molecules on the adsorption behavior of PMP and PMK were studied (Figure 8 and Table S4). Salvinolic acid B (SAB), rosmarinic acid (RA), danshensu (DSS), caffeic acid (CA), and ferulic acid (FA) were chosen as competitors of SAA for the adsorption selectivity experiment based on the mixed system. Considering the difference in the adsorption performance of the two adsorbents, the initial concentrations of each salvianolic acid for PMP were set as 1.0 mmol/L and those for PMK were set as 0.5 mmol/L. Both PMP and PMK showed stronger affinity for SAA in the light of higher *AE* and larger *K*_d_. Additionally, the *K*_d_ of SAA onto PMK was relatively higher than that of PMP, which may be related to the lower initial concentration or higher selectivity. Remarkably, we noticed that the *K*_d_ of PMK for SAA was over 7 times higher than those for other phenolic acids, which was more obvious than the difference of PMP for phenolic acids (4.6 times). When the effect of initial concentration was taken into consideration, the *CF* of PMK for SAA was still larger than that of PMP. As shown in Table S3, the *α* values for interfering compounds were far larger than 1. In addition, the *α* values of PMK were higher than those of PMP as well. The results indicated that the selectivity of PMK was higher than that of PMP. This phenomenon may be attributable to the combined effects of positive charge and the group density of phenylboronic acids. Moreover, PMP showed a higher adsorption capacity for each phenolic acid due to the presence of more phenylboronic acid groups.

### 2.7. Desorption Study

**Figure 9:**
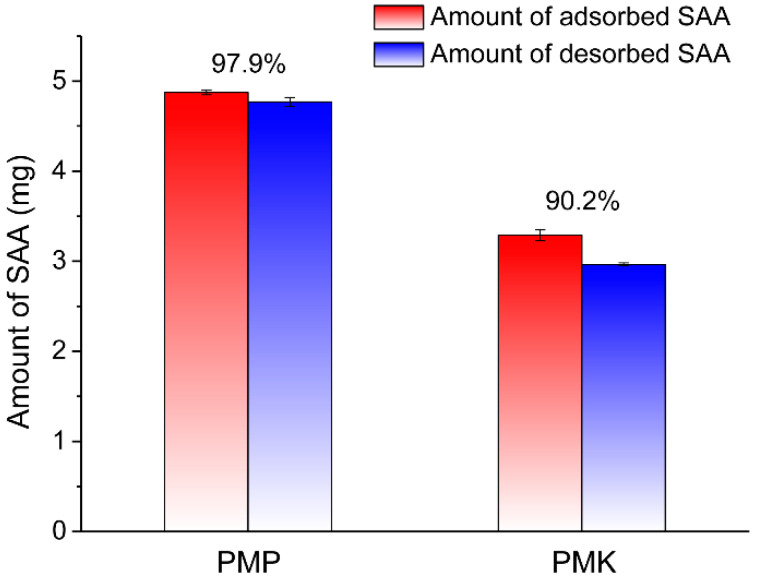
Desorption of SAA from PMP and PMK.

To assess the SAA recovery efficiency of the two adsorbents, a desorption study of SAA was conducted. In consideration of the acid-labile boronic acid ester group and poor adsorption performance at pH 1, HCl solution was selected as the desorbing agent in this study. As shown in Figure 9, 0.5 M HCl solution can desorb SAA from both PMP and PMK effectively. Additionally, 4.77 and 2.96 mg of SAA can be made with PMP and PMK under the condition of 10 mg of adsorbent and 10 mL of SAA solution (0.5 mg/mL), respectively. The desorption efficiency could reach over 90%. Remarkably, SAA can be eluted from PMP more effectively by HCl solution. The results may be ascribed to the higher percentage of phenylboronic acid groups in PMP.

## 3. Materials and Experiments

### 3.1. Materials

Montmorillonite (K-10, Al_2_O_9_Si_3_, 99%, 185.6 m^2^/g), branched polyethyleneimine (PEI, *M*_w_ = 3500 g/mol, 50 wt%), and 1-(3-dimethylaminopropyl)-3-ethylcarbodiimide (EDC, 98%) were all supplied by Shanghai Aladdin Biochemical Technology Co., Ltd. (Shanghai, China). Sodium carbonate was provided by Sinopharm Chemical Reagent Co. (Beijing, China), whereas 4-Carboxyphenylboronic acid (CPBA, 99%), *N*-hydroxysuccinimide (NHS, 99%), 3-aminopropyltriethoxysilane (KH550, 98%), and salvianolic acid A (SAA, 98%) were provided by Shanghai Yuanye Bio-Technology Co., Ltd. (Shanghai, China). The characterization methods are listed in Supporting Information in detail.

### 3.2. Preparation of Phenylboronic-Acid-Functionalized Montmorillonites with PEI (PMP)

MMT was initially sieved through a 200-mesh sieve to obtain micro-powder with a particle size of less than 75 μm. Then, to create Na-rich MMT, the as-prepared MMT powder (2.5 g) was combined with sodium carbonate (0.25 g) in 50 mL of deionized water for 5 h at 60 °C while being continuously stirred.

Then, to prepare MMT-PEI, the obtained Na-rich MMT was first reacted with intercalator PEI. Following this, phenylboronic-acid-functionalized montmorillonites containing PEI (PMP) were created by amidating the amino group of PEI with the carboxyl group of CPBA. Under ultrasound, 0.5 g of Na-rich MMT was dispersed in 50 mL of deionized water. After that, 0.5 mL of the PEI solution (50 wt%) was added into the system dropwise. Afterwards, MMT-PEI (0.2 g) was mixed with 50 mL of acetone solution containing CPBA (0.2963 g), EDC (0.4108 g), and NHS (1.0276 g). The mixture was stirred for 5 h at 35 °C to obtain PMP.

### 3.3. Preparation of Phenylboronic-Acid-Functionalized Montmorillonites with KH550 (PMK)

The as-prepared Na-rich MMT was reacted with KH550 to generate MMT-KH550. Then, phenylboronic-acid-functionalized montmorillonites with KH550 (PMK) were obtained via the reaction between CPBA and KH550. Specifically, Na-rich MMT (0.5 g) was disseminated in 50 mL of 75% ethanol solution using ultrasonic. The suspension was then treated with KH550 (0.5 g), and the mixture was refluxed for 10 h at 80 °C to produce MMT-KH550. Following that, 50 mL of acetone was used to dissolve CPBA (0.2963 g), EDC (0.4108 g), and NHS (1.0276 g). The as-prepared MMT-KH550 (0.2 g) was then mixed with the acetone solution and agitated for 5 h at 35 °C to obtain PMK.

### 3.4. Adsorption of Salvianolic Acid A by PMP and PMK

The adsorption experiment was typically carried out as follows: a polyethylene tube containing 1.0 g/L of functionalized MMT adsorbent and 0.5 mg/mL of SAA solution was shaken for a sufficient time at a rate of 200 rpm to reach adsorption equilibrium. PMP or PMK were then removed from the suspension using a filter head (0.22 μm). At a wavelength of 286 nm, a UV-vis spectrometer was used to measure the SAA concentration. The equilibrium adsorption amount (*q*_e_), adsorption efficiency (*AE*), distribution ratio (*K*_d_), separation factor (*α*), and concentration factor (*CF*) of SAA can be calculated using Formulas (1)–(5), respectively [12,39]:

(1) $$q_{e}=(c_{0}-c_{e})\times \frac{V}{m}$$

(2) $$AE\;(\%)=\frac{c_{0}-c_{e}}{c_{e}}\times 100$$

(3) $$K_{d}=\frac{c_{0}-c_{e}}{c_{e}}\times \frac{V}{m}$$

(4) $$\alpha =\frac{K_{d}(\mathrm{SAA})}{K_{d}(\mathrm{Competing}\;\mathrm{compound})}$$

(5) $$CF=\frac{q_{e}}{c_{0}}$$

where *c*_0_ and *c*_e_ (mg/L) represent the concentrations of SAA in the solution before and after adsorption, respectively. *m* (g) stands for the weight of the dry adsorbent, and *V* (L) is the volume of the SAA solution.

### 3.5. Desorption Study

The desorption experiment was conducted as follows: 10 mg of functionalized MMT adsorbent was added into 10 of SAA solution (0.5 mg/mL). After saturated adsorption, the adsorbent–SAA complex was separated from the system and the concentration of SAA was measured to calculate the amount of adsorbed SAA. Then, the adsorbent–SAA complex was eluted with 10 mL of HCl solution (0.5 M) at room temperature for 5 h. Subsequently, the concentration of SAA in elution was measured to calculate the amount of desorbed SAA.

## 4. Conclusions

In summary, the structures and SAA adsorption performances of phenylboronic-acid-functionalized montmorillonites with different intercalators including cationic polyelectrolyte (PEI) and amino-terminated silane (KH550) were investigated. The type of intercalator had an impact on the structural and morphological characteristics. Amino-terminated silane demonstrated a more thorough intercalation effect, meaning that the obtained MMT possessed a 2D structure with a higher specific surface area. According to the results of EDX and zeta potential, however, MMT intercalated with cationic polyelectrolyte had more active sites for modification and adsorption. Since PMP had more adsorption sites, its adsorbent dose was lower than that of PMK. The effect of pH on PMP adsorption for SAA was more noticeable than for PMK. Additionally, PMK showed a faster adsorption kinetic than PMP due to a higher surface area and fewer functional groups, whereas the intra-particle diffusion process of PMP took less time. The endothermic process of PMP is more obvious, as it accounts for a higher percentage of chemical reactions during the adsorption process. For PMP, a larger adsorption capacity can be achieved more easily by raising the adsorption temperature. The adsorption capacity of PMP for SAA from the Langmuir isotherm model could reach 593.2 mg/g, which was larger than that of PMK (421.2 mg/g), owing to the higher content of phenylboronic acid groups. A greater adsorption selectivity for SAA was demonstrated by PMK as a result of the combined effects of the density of positive charge and functional group. SAA can be desorbed from PMP by 0.5 M HCl solution more effectively. This comparative SAA adsorption study demonstrated that functionalized montmorillonites intercalated with PEI performed better in terms of adsorption. Nevertheless, after dealing with the density of function groups, cationic silane might be a viable intercalator. This work provides an alternative suggestion for the preparation of functional montmorillonite from the standpoint of the selection of an intercalator.

## Figures and Tables

**Table 1:** BET parameters of Na-rich MMT, PMP, and PMK.

Samples	BET Surface Area (m^2^/g)	Pore Diameter (nm)	Pore Volume (cm^3^/g)
Na-rich MMT	201.9	7.4	0.375
PMP	55.4	11.9	0.166
PMK	69.9	8.8	0.153

## Data Availability

Research data not available in the manuscript can be obtained from the corresponding author upon reasonable request via email.

## Supplementary Materials

The following supporting information can be downloaded at: https://www.mdpi.com/article/10.3390/molecules28135244/s1, Figure S1: SEM image of MMT; Figure S2: Nitrogen adsorption-desorption isotherms and pore size distributions of materials; Figure S3: Comparison of SAA uptake of different adsorbents. Table S1: Adsorption kinetic parameters for adsorbents; Table S2: Thermodynamic parameters for the adsorption of SAA by PMP and PMK; Table S3: Langmuir isotherm parameters for adsorbents; Table S4: Selective adsorption of SAA on PMP and PMK. References [30,31,32,40,41] are cited in the supplementary materials.
