# Supplementary material for: Comparison of Salvianolic Acid A Adsorption by Phenylboronic-Acid-Functionalized Montmorillonites with Different Intercalators
Source: Molecules. 2023 Jul 6;28(13):5244. doi: 10.3390/molecules28135244 (PMC10343553; doi:10.3390/molecules28135244)
Supplement: Supplementary file 1 [file molecules-28-05244-s001.zip › molecules-2465534-supplementary.pdf]

Supplementary Materials

Comparison of Salvianolic Acid A Adsorption by Phenylboronic-Acid-Functionalized Montmorillonites with Different Intercalators

Jun Qian [1,†], Jiajia Su [1,†], Weihuan Zeng [1], Yue Wang [1], Yingyuan Hu [2] and Guoyin Kai [1,*]

[1] Zhejiang Provincial TCM Key Laboratory of Chinese Medicine Resource Innovation and Transformation, Zhejiang Provincial International S&T Cooperation Base for Active Ingredients of Medicinal and Edible Plants and Health, School of Pharmaceutical Sciences, Jinhua Academy, Zhejiang Chinese Medical University, Hangzhou 311402, China; qianjun_1116@163.com (J.Q.); s15951135946@163.com (J.S.); z1437143688@126.com (W.Z.); wy102799@163.com (Y.W.)

[2] School of Chemistry and Life Sciences, Suzhou University of Science and Technology, Suzhou 215009, China; huyy@usts.edu.cn

* Correspondence: guoyinkai1@126.com; Tel.: +86-571-86633328; Fax: +86-571-86633171

† These authors contributed equally to this work.

Characterization methods

Field-emitting scanning electron microscopy (SEM) images were captured using a Hitachi SU8010 microscope. The Bruker D8 Advance diffractometer was used to acquire data on powder X-ray diffraction (XRD) with a 0.02° step in the 2 theta angle range of 2 to 30°. The data acquisition time was 0.5 s. The ASAP 2460 V3.01 H analyzer was used to calculate surface areas and pore size distributions. Malvern Zetasizer with irradiation (He-Ne laser, 632.8 nm) was used to determine the material's zeta potentials. A Hitachi S-3000N E-1010 scanning electron microscope was used to conduct the energy-dispersive X-ray (EDX) examination. Thermo ESCALAB 250Xi XPS was used to conduct X-ray photoelectron spectroscopy (XPS). Shimadzu UV-2550 spectrometer and Agilent 1260 Series high-performance liquid chromatography (HPLC) were used to determine the concentration of SAA.

Calculation of interlayer space

The Bragg equation can be used to calculate the relevant interlayer distance: $2d \sin \theta = n \lambda$ where d stands for interlayer distance, θ stands for the angle between the corresponding crystal surface and the incident X-ray, n (1) denotes a diffraction series, and λ (1.54056 Å) is the wavelength of the incident X-ray.

Adsorption kinetics

Pseudo-first-order kinetic equation describes the relationship between the adsorption rate and adsorption amount q_t at time t by the following equation [1]

$$q_t = q_e (1 - e^{-k_1 t}) \qquad \text{(S1)}$$

where k_1 (min^{-1}) is the pseudo-first-order kinetic constant and q_e is equilibrium capacity.

The pseudo-second-order model is expressed by [2, 3]

$$q_t = q_e^2 k_2 t / (1 + q_e k_2 t) \qquad \text{(S2)}$$

where k_2 (g/mmol/min) is the second order rate constant.

Adsorption thermodynamics [4]

$K_c = q_e / c_e$ (S3)

$\Delta G^o = -RT\ln K_c$ (S4)

$\ln K_c = \Delta S^o / R - \Delta H^o / (RT)$ (S5)

where R and T are the ideal gas constant (8.314 J/mol/K) and the absolute temperature, respectively. ΔH^o and ΔS^o can be obtained from the slope and intercept of the plot of $\ln K_c$ versus $1/T$.

Adsorption isotherms

Langmuir model is given as Equation (S6), which describes monolayer adsorption based on the assumption that all the adsorption sites have equal affinity, and that desorption at one site doesn't affect an adjacent site [5].

$q_e = q_{max} b c_e / (1 + b c_e)$ (S6)

where q_{max} (mmol/g) is the maximum adsorption amount (i.e. adsorption capacity), and b (L/mmol) is the Langmuir constant.

Table S1. Adsorption kinetic parameters for adsorbents (c_0 = 0.5 mg/mL, pH = 3, V = 3 mL, $m_{adosrbent}$ = 3 mg, T = 298.15K).

Adsorbent	$q_{e, exp}$ (mg/g)	Pseudo-first order			Pseudo-second order		
		k_1 (min^{-1})	$q_{e, cal}$ (mg/g)	R^2	k_2 (g/mg/min)	$q_{e, cal}$ (mg/g)	R^2
PMP	453.52	0.3279	436.80	0.9845	0.0017	448.09	0.9971
PMK	259.31	0.3207	247.71	0.9876	0.0030	253.92	0.9962

Table S2. Thermodynamic parameters for the adsorption of SAA by PMP and PMK (c_0 = 0.5 mg/mL, pH = 3, V = 3 mL, $m_{adosrbent}$ = 3 mg).

Adsorbents	ΔH^o (kJ/mol)	ΔS^o (J/mol/K)	ΔG^o (kJ/mol)			
			298.15 K	308.15 K	318.15 K	328.15 K
PMP	28.91	152.24	-16.68	-17.54	-19.70	-21.06
PMK	10.62	91.42	-16.50	-17.70	-18.53	-19.25

Table S3. Langmuir isotherm parameters for adsorbents.

Adsorbents	Langmuir isotherm model		
	q_{max} (mg/g)	b (L/mg)	R^2
MMT	197.2	1.52	0.93
PMP	593.2	4.94	0.98
PMK	421.2	6.05	0.99

Table S4. Selective adsorption of SAA on PMP and PMK (PMP: c_0 for each salvianolic acid = 1.0 mmol/L, pH = 3, V = 3 mL, $m_{adosrbent}$ = 3 mg, T = 298.15K; PMK: c_0 for each salvianolic acid = 0.5 mmol/L, pH = 3, V = 3 mL, $m_{adosrbent}$ = 3 mg, T = 298.15K).

Compounds	AE (%)		K_d (L/g)		q_e (mmol/g)		CF (L/g)		α	
	PMP	PMK	PMP	PMK	PMP	PMK	PMP	PMK	PMP	PMK
SAA	92.73	96.31	12.50	26.12	0.9273	0.4816	0.9273	0.9649	1.00	1.00
SAB	73.46	78.41	2.71	3.631	0.7346	0.3920	0.7346	0.7822	4.608	7.192
RA	39.29	27.63	0.635	0.382	0.3929	0.1381	0.3929	0.2761	19.70	68.42
DSS	6.629	0.374	0.070	0.004	0.0663	0.0019	0.0706	0	179.6	6962
CA	8.156	0	0.087	0	0.0816	0	0.0816	0	143.6	/
FA	7.057	0	0.074	0	0.0706	0	0.0663	0.0038	168.0	/

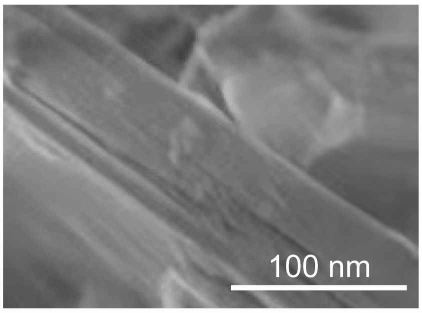

Figure S1. SEM image of MMT, scale bar: 100 nm.

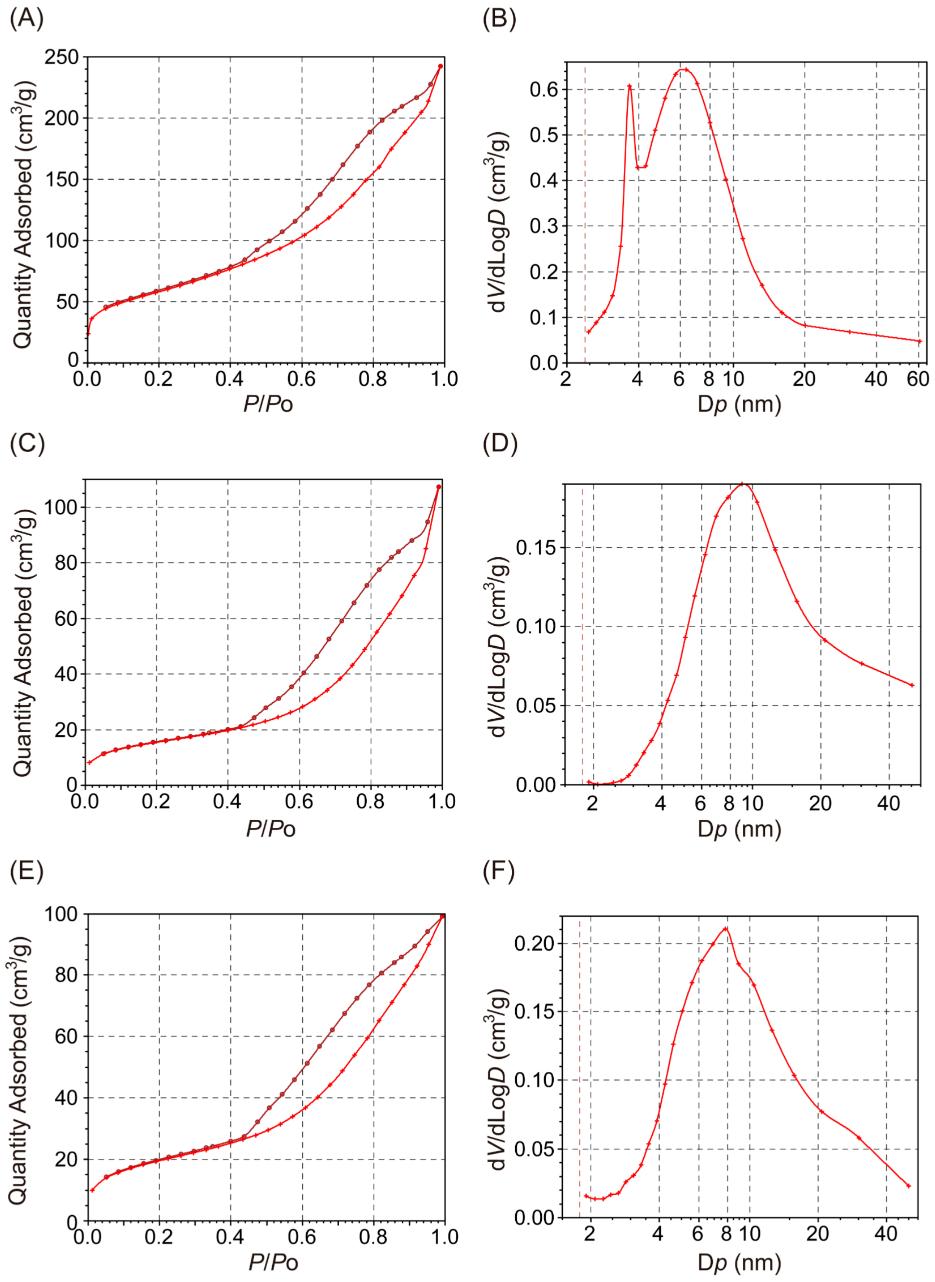

Figure S2. (A, C, E) Nitrogen adsorption-desorption isotherms of Na-rich MMT, PMP and PMK, respectively. (B, D, F) Pore size distributions of Na-rich MMT, PMP and PMK, respectively.

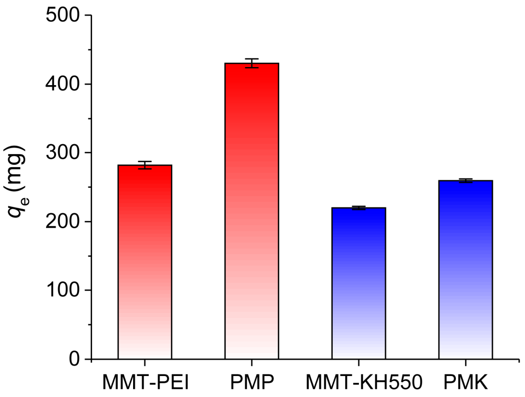

Figure S3. Comparison of SAA uptake of MMT-PEI, PMP, MMT-KH550 and PMK (c_0 = 0.5 mg/mL, pH = 3, V = 3 mL, $m_{\text{adosrbent}}$ = 3 mg, T = 298.15K).

References

[1] Lagergren S. Zur theorie der sogenannten adsorption gelöster stoffe. K Sven Vetenskapsakad, Handl, Band 24 1898; 4: 1-39.

[2] Ho Y-S, McKay G. Sorption of dye from aqueous solution by peat. Chemical Engineering Journal 1998; 70: 115-24.

[3] Ho YS. Review of second-order models for adsorption systems. Journal of Hazardous Materials 2006; 136: 681-9.

[4] Sarı A, Tuzen M, Soylak M. Adsorption of Pb (II) and Cr (III) from aqueous solution on celtek clay. Journal of Hazardous Materials 2007; 144: 41-6.

[5] Langmuir I. The constitution and fundamental properties of solids and liquids Part I Solids. Journal of the American Chemical Society 1916; 38: 2221-95.
